# Conduction Disorders during Sinus Rhythm in Relation to Atrial Fibrillation Persistence

**DOI:** 10.3390/jcm10132846

**Published:** 2021-06-27

**Authors:** Willemijn F. B. van der Does, Annejet Heida, Lisette J. M. E. van der Does, Ad J. J. C. Bogers, Natasja M. S. de Groot

**Affiliations:** 1 Department of Cardiology, Erasmus Medical Center, 3015 CN Rotterdam, The Netherlands; w.vanderdoes@erasmusmc.nl (W.F.B.v.d.D.); a.heida@erasmusmc.nl (A.H.); j.vanderdoes@erasmusmc.nl (L.J.M.E.v.d.D.); 2 Department of Cardiothoracic Surgery, Erasmus Medical Center, 3015 CN Rotterdam, The Netherlands; a.j.j.c.bogers@erasmusmc.nl

**Keywords:** atrial fibrillation, conduction disturbances, high-resolution mapping, AF persistence

## Abstract

Classification of atrial fibrillation (AF) is currently based on clinical characteristics. However, classifying AF using an objective electrophysiological parameter would be more desirable. The aim of this study was to quantify parameters of atrial conduction during sinus rhythm (SR) using an intra-operative high-resolution epicardial mapping approach and to relate these parameters to clinical classifications of AF. Patients were divided according to the standard clinical classification and spontaneous termination of AF episodes. The HATCH score, a score predictive of AF progression, was calculated, and surface ECGs were evaluated for signs of interatrial block. Conduction disorders mainly differed at Bachmann’s bundle (BB). Activation time (AT) at BB was longer in persistent AF patients (AT-BB: 75 (53–92) ms vs. 55 (40–76) ms, *p* = 0.017), patients without spontaneous termination of AF episodes (AT-BB: 53.5 (39.6–75.8) ms vs. 72.0 (49.6–80.8) ms, *p* = 0.009) and in patients with a P-wave duration ≥ 120 ms (64.3 (52.3–93.0) ms vs. 50.5 (39.6–56.6) ms, *p* = 0.014). HATCH scores also correlated positively to AT-BB (rho 0.326, *p* = 0.029). However, discriminatory values of electrophysiological parameters, as calculated using ROC-curves, were limited. These results may reflect shortcomings of clinical classifications and further research is needed to establish an objective substrate-based classification of AF.

## 1. Introduction

Atrial fibrillation (AF) is classified in the ESC guidelines according to the clinical presentation and duration of AF episodes. Pulmonary vein isolation is recommended for paroxysmal AF patients with symptomatic recurrences of AF on antiarrhythmic drug therapy; however, late recurrences of AF after ablation are common [1,2]. The unpredictable, moderate success rate of pulmonary vein isolation is not only caused by reconduction but is also likely the result of differences in severity and extent of electropathology in patients with the same AF subtype [3,4,5]. In the latter case, a different classification based on AF-related electropathology should be considered to select patients who will benefit from pulmonary vein isolation.

Previously, mapping studies have shown differences in conduction characteristics between paroxysmal and persistent AF patients. During pacing, conduction slowing was more pronounced in the left atrium (LA) and Bachmann’s bundle (BB) in patients with persistent AF, compared to patients with paroxysmal AF [6,7]. During sinus rhythm (SR), a longer activation time of the LA was found in persistent AF patients compared to paroxysmal AF patients [8,9,10]. In contrast, in the pulmonary vein area (PVA) no relation was found between severity of conduction disorders in SR and AF subtypes [11].

These contradictory results raise the question whether differences between paroxysmal and persistent AF are confined to specific anatomical regions. It is unknown whether there are discriminatory differences in characteristics of conduction disorders during SR in both atria between paroxysmal and persistent AF patients. If differences are present, an objective classification based on electropathology could be established.

The goal of this study is, therefore, to compare conduction disorders during SR between patients with paroxysmal and persistent AF using a high resolution epicardial mapping approach covering the right atrium (RA), BB, LA and the PVA. As the standard classification of AF might be limited in its discriminative ability, we also compare conduction disorders between patients with or without spontaneous termination of AF episodes and between patients with or without ECG characteristics identifying interatrial block. We also correlate conduction characteristics to the HATCH score, a score predicting progression of paroxysmal AF [12].

## 2. Experimental Section

### 2.1. Study Population

Patients (≥ 18 years) undergoing elective cardiac surgery for either coronary bypass grafting, aortic or mitral valve surgery or a combination of these procedures were included. This study was approved by the Medical Ethical Committee in the Erasmus Medical Center (MEC 2010-054 and MEC 2014-393) and follows the declaration of Helsinki principles. Written informed consent was obtained from all patients. Clinical data was collected from electronic records; patients with a history of AF were selected for this study. In line with the ESC guidelines, patients with documentation (ECG, ECG description) of AF episodes up to 7 days, or with AF episodes cardioverted within 7 days were classified as paroxysmal AF. Patients with documentation of AF episodes longer than 7 days or longer than a year were classified as persistent and long-standing persistent AF, respectively. In patients with paroxysmal AF, the HATCH score (hypertension, age (>75 years), transient ischemic attack or stroke, chronic obstructive pulmonary disease and heart failure) was calculated. Additionally, patients were subdivided in 2 groups according to whether AF episodes pre-operatively terminated spontaneously or required intervention (electrical or chemical cardioversion). In patients presenting with SR before surgery, ECGs were evaluated for signs of advanced interatrial block (a-IAB) and the duration of the P-wave as described by Bayes de Luna et al. [13]

### 2.2. Mapping Procedure

Epicardial mapping was performed during open heart surgery, prior to initiation of extracorporal circulation. A steel wire was fixed into subcutaneous tissue serving as an indifferent electrode. A bipolar epicardial pacemaker wire, stitched into the right atrium free wall, served as a reference electrode. Mapping was performed during SR with either a custom-made 128-unipolar electrode array or a 192-unipolar electrode array, inter-electrode distances of both arrays were 2 mm.

Mapping was conducted by shifting the electrode array consecutively over the right atrium (RA) to BB and the LA including PVA, as previously described in detail [14]. This mapping method, including positioning of the reference electrode, has been standardized and used for more than a decade by a group of dedicated cardiothoracic surgeons who perform these mapping procedures. If the rhythm at the onset of the mapping procedure was AF, electrical cardioversion was performed to restore SR. At every site, five seconds of SR were recorded, including a surface ECG, bipolar reference and a calibration signal of 2 mV and 1000 ms. Recordings were amplified with gain 1000, sampled with a rate of 1 kHz, filtered with bandwidth 0.5–400 Hz, analogue-to-digital converted (16 bits) and stored on hard disk.

### 2.3. Data Processing

Using dedicated mapping software, unipolar electrograms were analyzed. As unipolar electrograms provide more detailed information, especially in our high-resolution mapping setting, we chose to use unipolar instead of bipolar electrograms. The steepest part of the negative deflection was automatically annotated if the amplitude exceeded the noise level in the channel with a probability of 99.95%. Local activation times (LAT) of these annotations were translated into an activation map for every sinus beat. All markings were manually verified. Electrogram files were excluded when deflections were marked in less than 40% of the mapping area due to recordings of poor quality (low signal to noise ratios).

**Figure 1:**
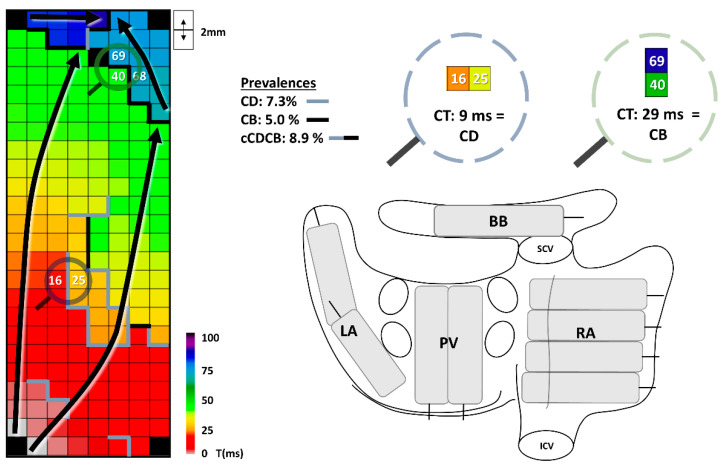
Conduction delay and block. The left panel shows a color-coded activation map of BB with conduction disturbances and arrows showing the direction of wavefront propagation. The upper right panel shows the corresponding prevalences of conduction delay (CD), conduction block (CB) and continuous conduction delay and block (cCDCB). CT: conduction time; the time difference between two electrodes. The lower right panel shows an overview of the mapping locations on a posterior view of the heart. BB: Bachmann’s bundle; ICV: internal vena cava; LA: left atrium; PVA: pulmonary vein area; SCV: superior vena cava; RA: right atrium.

For each electrode the conduction time (CT) was calculated as the difference in LAT with the bordering right and lower electrodes, as illustrated in Figure 1. As in prior mapping studies, conduction delay (CD) was defined as CTs >6 and <12 ms, conduction block (CB) was defined as CTs >11 ms. These cutoffs for CB were determined, as explained in previous work, based on the slowest conduction during longitudinal propagation [15]. When CB lines connected with CD lines, this was labeled as a continuous conduction delay and block (cCDCB) line. These parameters are shown in detail in the upper right panel in Figure 1. All conduction parameters were calculated per mapping site (e.g., LA1, LA2) and then translated to an average, maximum or sum for the corresponding location (e.g., LA). Total activation time (TAT) and activation time (AT) for every location were determined by comparing the first and last activation of the corresponding mapping sites in relation to the bipolar reference electrode. At BB, as fiber direction in this location is known, we evaluated the direction of CB lines as being mostly perpendicular to, in parallel with, or diagonal on the fiber direction. If a perpendicular CB line completely isolated the right and left side of BB, this was scored as an isolating CB line.

### 2.4. Statistical Analysis

Normally distributed data are described by mean ± standard deviation, skewed data by median (interquartile range) and categorical data by absolute number (percentage). Differences between groups were calculated with a student’s *t*-test or Mann–Whitney U. When data was categorical, chi-square testing or an exact test was performed if appropriate. Correlations between HATCH score and electrophysiological parameters were evaluated using Spearman rank correlation. Multiple linear regression was performed to correct for confounding in continuous data, using a log transformation with the parameters AT-BB and AT-LA as the criteria for linear regression were violated. We constructed ROC-curves and evaluated the area under the curve (AUC) to determine the discriminatory value of various electrophysiological parameters. A *p*-value <0.05 was considered statistically significant. All statistical analyses were performed with IBM SPSS statistics for Windows, version 25 (IBM Corp., Armonk, NY, USA).

## 3. Results

### 3.1. Patient Characteristics

The study population consisted of 71 patients (age 72 ± 7 years, 28 (39%) female); baseline characteristics are shown in Table 1 with patients divided according to clinical classification. Patients had either paroxysmal (*n* = 47, 66%) or persistent (*n* = 24, 34%) AF, including three patients with long-standing persistent AF. Indications for cardiac surgery were coronary artery bypass grafting (*n* = 20, 28%), aortic or mitral valve surgery or a combination of valvular and bypass surgery (*n* = 51, 72%). There were no significant differences in baseline characteristics between patients with paroxysmal and persistent AF. Cardioversion before mapping was necessary in three (6.4%) patients from the paroxysmal AF group, and 17 (70.8%) patients from the persistent AF group.

The duration of the longest AF episode in patients with paroxysmal AF was less than 1 day for 18 (38.2%) patients and between 1–6 days for 10 patients (21.3%). For the remaining patients classified as paroxysmal AF (*n* = 19, 40.4%) there was no information on duration of AF episodes. In the persistent AF group, the longest AF episode was 3 (1–5.5) months, ranging between 2 weeks and 2 years. SR cycle length was 874.1 (760.8–1012.8) ms in the paroxysmal AF group and 857.7 (751.4–1005.6) ms in the persistent AF group (*p* = 0.498).

### 3.2. Conduction Disorders in Paroxysmal and Persistent AF Patients

The prevalence of CB in the entire atria in the paroxysmal AF group ranged between 0.5% and 8.7% and between 0.4% and 7.0% in the persistent AF group. As demonstrated in Supplemental Table S1, these large ranges were present at all locations. For all locations, there were no differences in prevalences of CD, CB and cCDCB between the paroxysmal and persistent AF patients. A higher number of cCDCB lines was found at BB in the persistent AF group (2 (1–3) vs. 3 (2–4), *p* = 0.040). However, there were no differences in length of (longest) lines of CD, CB, or cCDCB between the paroxysmal and persistent AF group in any location.

**Figure 2:**
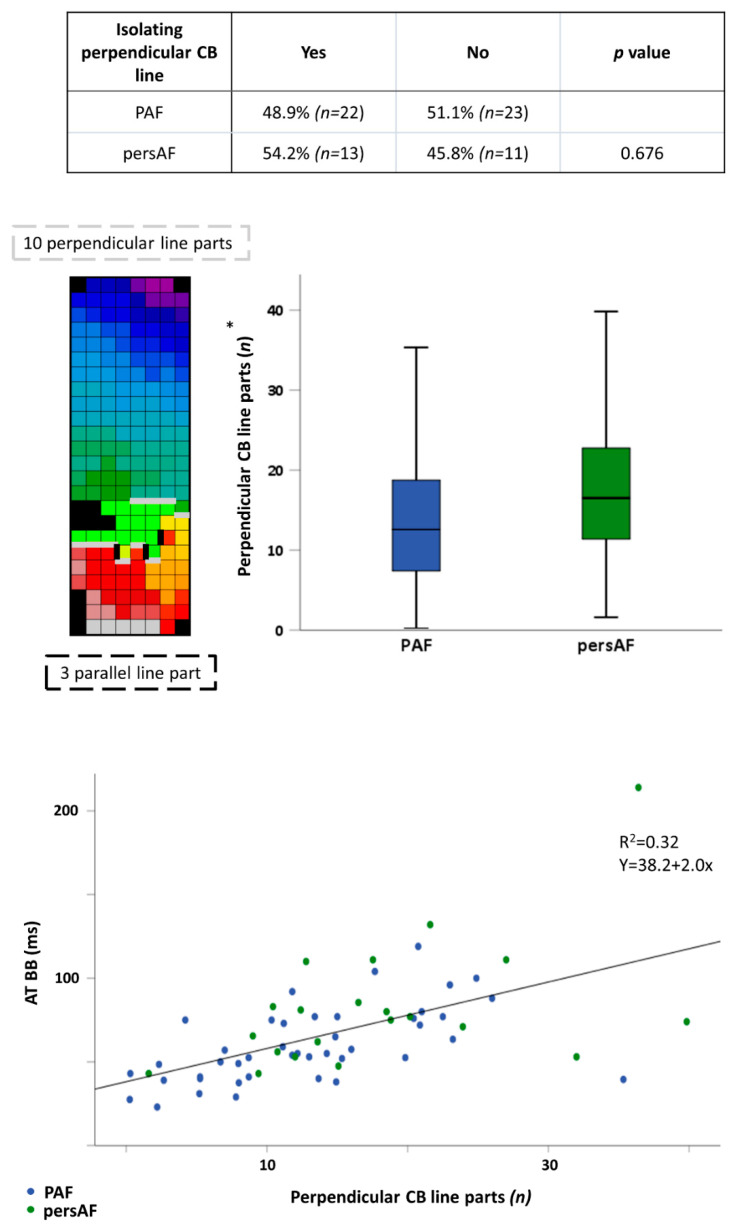
Paroxysmal vs. persistent AF patients: conduction disorders at Bachmann’s Bundle. The table in the upper panel shows percentages of PAF and persAF patients with an isolating perpendicularly orientated CB line. The left part of the middle panel illustrates how the number of parallel and perpendicular orientated CB line parts was determined. An activation map is depicted with gray perpendicular CB line parts and black parallel CB line parts. The right part of the middle panel shows the difference in perpendicular CB line parts between PAF and persAF patients. The scatterplot in the lower panel depicts the relation between amount of perpendicular CB line parts and AT at BB in all patients. BB: Bachmann’s bundle; CB: conduction block; PAF: paroxysmal AF; persAF: persistent AF; TAT: total activation time; * *p* = 0.045.

Conduction disorders particularly occurred at BB. Perpendicular orientation of the longest CB line at BB was more frequently present in patients with persistent AF (*n* = 19, 82.6%), than in patients with paroxysmal AF (*n* = 22, 52.4%), *p* = 0.028. There was no difference in prevalence of isolating CB lines (upper panel Figure 2). The exact number of perpendicularly orientated CB line parts was evaluated as explained in the left part of the middle panel of Figure 2. Patients with persistent AF had more perpendicularly orientated parts of CB line than paroxysmal AF patients (middle panel Figure 2; PAF: 13.0 ± 7.7, persAF: 18.3 ± 10.0, *p* = 0.045). The number of perpendicularly orientated parts of CB was correlated with AT of BB (lower panel Figure 4; rho 0.605, *p* < 0.0005).

**Figure 3:**
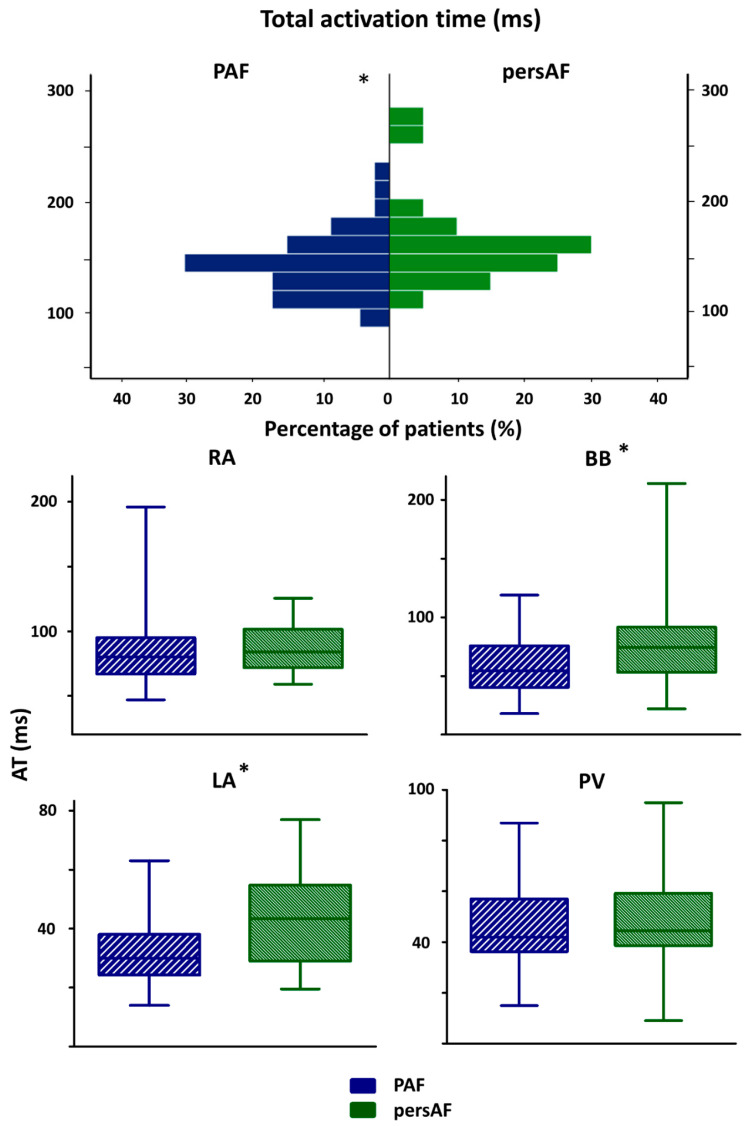
Paroxysmal vs. persistent AF patients: activation times. The upper panel shows the distribution of total activation time for the PAF group and persAF group. The lower panel depicts ATs for each location separately in the PAF and persAF group. BB: Bachmann’s bundle; LA: left atrium; PVA: pulmonary vein area; PA: paroxysmal AF; persAF: persistent AF; RA: right atrium; AT: activation time; * total activation time; *p* = 0.034, BB: *p* = 0.017, LA: *p* = 0.010.

As demonstrated in Figure 3, TAT in the persistent AF group (156 (135–172) ms) was longer than TAT in the paroxysmal AF group (137 (122–154) ms), *p* = 0.034. This distinction was caused by a longer AT-LA (45 (29–55) ms vs. 30 (25–39) ms, *p* = 0.010) and AT-BB (75 (53–92) ms vs. 55 (40–76) ms, *p* = 0.017) in the persistent AF group.

To exclude a confounding effect of clinical parameters, we performed a multivariate analysis for TAT, AT-BB and AT-LA (Supplemental Table S2). Persistent AF remained an independent predictor of a longer TAT (*p* = 0.031) and AT-BB (*p* = 0.008), but not of a longer AT-LA (*p* = 0.286). However, the multivariate analyses did not include all patients, as LAVI was not available for every patient. The multivariate analyses showed female gender and age as independent predictors for a longer AT-BB.

**Figure 4:**
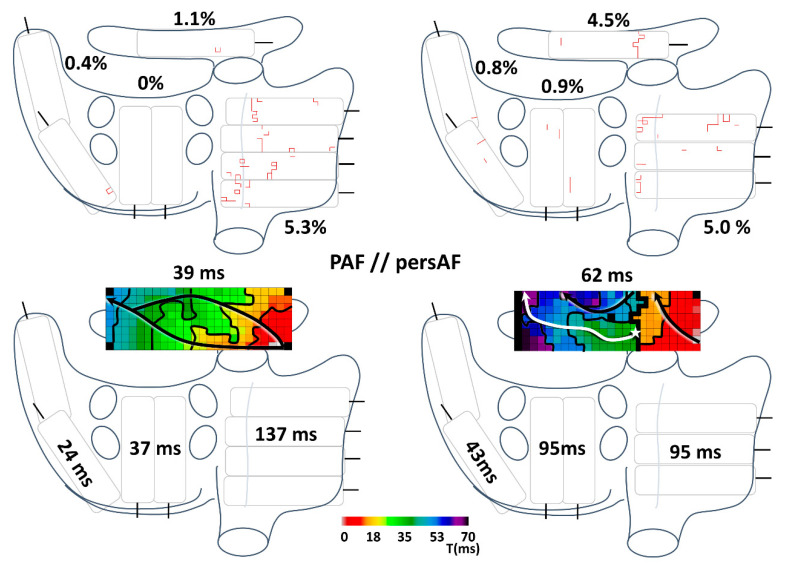
Typical paroxysmal and persistent AF patient. The distribution of CB prevalence (upper panel) and AT (lower panel) is seen in a PAF (left) and a persAF patient (right). In the lower panel, two activation maps are demonstrated at BB with 10 ms isochrones, arrows showing the direction of wavefront propagation and thick black lines indicating CB area. The paroxysmal AF patient, 69 years old, was diagnosed 3 months before he underwent mitral valve surgery. He had been experiencing palpitations for minutes to hours at a time and AF occurred during a cardiac exercise stress test while he experienced similar palpitations. The persistent AF patient, 64 years old, was diagnosed with AF 10 months before he underwent mitral valve surgery. As duration of AF was unknown at the time of diagnosis, an electrocardioversion was scheduled 2 months later, which was successful. The patient had two more AF episodes lasting longer than 7 days requiring electrocardioversion before surgery, which restored SR in both cases. CB: conduction block; PAF: paroxysmal AF; persAF: persistent AF; AT: activation time.

An example of the distribution of the prevalence of CB and AT in the atria of a typical paroxysmal and typical persistent AF patient is demonstrated in Figure 4. The paroxysmal AF patient was diagnosed 3 months before surgery and the persistent AF patient was diagnosed 10 months before surgery. Both underwent mitral valve surgery. In the upper panel of Figure 4, comparable prevalences of CB are seen in the paroxysmal and persistent AF patient in all areas except BB. In the patient with persistent AF, an isolating CB line in BB is seen; a thick black CB line which connects the lower and upper side of the mapping array. Most CB lines in the RA in this figure, especially apparent in the paroxysmal AF patient, are found in the crista terminalis area. In the lower panel of Figure 4, the AT for every location is depicted. The AT of LA, PVA and BB are longer in the persistent AF patient, while the AT of RA is longer in the paroxysmal AF patient.

### 3.3. Conduction Disorders in Relation to Spontaneous Termination of AF Episodes

Thirty-nine patients had spontaneous termination of AF episodes (ST group) and 32 patients had AF episodes that did not terminate spontaneously (NST group).

Patients without spontaneous termination of AF episodes had a prolonged TAT (ST: 134.0 (115.8–153.3) ms vs. NST: 152.5 (136.4–165.1) ms, *p* = 0.033) and AT-BB (ST: 53.5 (39.6–75.8) ms, NST: 72.0 (49.6–80.8) ms, ST *p* = 0.009). Other conduction characteristics did not differ between the groups.

### 3.4. Conduction Disorders in Relation to P-Wave Duration and a-IAB

In 48 (67.6%) patients, of whom six patients with persistent AF, an ECG was available for evaluation of P-wave duration and a-IAB. In 27 patients (56.3%), P-wave duration was ≥ 120 ms and five patients (11.1%) met the criteria of an a-IAB.

Patients with a P-wave duration ≥ 120 ms had a significantly higher prevalence of CB and cCDCB at BB (CB: 8.5 ± 5.7% vs. 4.3 ± 2.5%, *p* = 0.001; cCDCB: 10.8 ± 7.6% vs. 6.2 ± 4.2%, *p* = 0.012). This was due to a prolongation of the longest CB and cCDCB lines at BB in these patients (CB: 32(22–40) mm vs. 20 (14–28) mm, *p* = 0.014; cCDCB: 52 (30–76) mm vs. 34 (20–44) mm, *p* = 0.026). At the PVA, the length of cCDCB lines was longer in patients with a P-wave duration ≥ 120 ms (15.7 (12.1–23.8) mm vs. 13.5 (10.1–14.9) mm, *p* = 0.036). Furthermore, AT-RA and AT-BB were longer in patients with a P-wave duration ≥ 120 ms (AT-RA: 89.0 (77.9–104.5) ms vs. 75.0 (64.0–88.0) ms, *p* = 0.010; AT-BB: 64.3 (52.3–93.0) ms vs. 50.5 (39.6–56.6) ms, *p* = 0.004).

Patients with a-IAB also had a higher prevalence of CB at BB (12.7 (6.8–17.8)% vs. 5.1 (3.2–8.4)%, *p* = 0.028), which was caused by a prolongation of the longest CB line at BB in these patients (40 (30–77) mm vs. 26 (16–34) mm, *p* = 0.018). The maximum CT at BB was longer in patients with a-IAB (80 (55–99) ms vs. 32 (23.5–27) ms, *p* < 0.0005). Remarkably, the prevalence of CD at the LA was lower in patients with a-IAB (0.9 (0.3–1.7)% vs. 2.7 (1.6–3.8)%, *p* = 0.006). At the PVA, the prevalence of CD was higher in patients with a-IAB (5.8 (5.5–6.2)% vs. 3.4 (1.8–5.3)%, *p* < 0.0005). Furthermore, patients with a-IAB had a longer AT-RA (117.5 (101.8–175.0) ms vs. 80.3 (66.6–93.1) ms, *p* < 0.0005), AT-BB (92.0 (80.5–115.0) ms vs. 53.0 (41.0–63.5) ms, *p* = 0.002), AT-PV (63.0 (51.0–79.8) ms vs. 41.0 [37.0–53.0 ms, *p* = 0.005) and TAT (213.0 (178.0–240.3) ms vs. 137.0 (119.1–152.8) ms, *p* < 0.0005). However, these comparisons are based on only five patients with a-IAB on ECG.

### 3.5. Discriminatory Value of Electropathology

The most optimal ROC-curve for paroxysmal vs. persistent AF patients, patients with vs. without spontaneous termination and patients with vs. without a P-wave duration ≥ 120 ms was of AT-BB (AUC 0.681 [0.544–0.818], TAT (AUC 0.689 [0.562–0.817]) and again AT-BB (AUC 0.749 [0.608–0.890]), respectively. Thus, none of these conduction parameters were clearly discriminatory. We did not construct ROC-curves for patients with vs. without a-IAB, as only five patients met the criteria for a-IAB. The AUC values for all ROC-curves are depicted in the Supplemental Table S3.

### 3.6. Correlation of Conduction Parameters with HATCH Score

The median HATCH score was measured in all paroxysmal AF patients and was 2 [1,2,3]; only three patients had a score higher than 4. The HATCH score correlated with CB prevalence at BB (rho 0.326, *p* = 0.029) and AT-BB (rho 0.378, *p* = 0.011).

## 4. Discussion

### 4.1. Main Findings

A higher number of conduction disorders was mainly found at BB in patients with persistent AF, without spontaneous termination of AF, with a P-wave duration ≥ 120 ms or with a higher HATCH score undergoing cardiac surgery. In patients with persistent AF, there was a higher number of cCDCB lines and perpendicularly orientated CB lines. Consequently, both AT-BB and TAT were prolonged.

However, the AUCs of the ROC-curves using electrophysiological parameters to distinguish AF subtypes were only moderately discriminatory.

### 4.2. Clinical Classification of AF

Prior studies have demonstrated that persistent AF patients can regress to paroxysmal AF and even the AF-burden cannot distinguish paroxysmal from persistent AF [16,17]. Shortcomings of the standard clinical classification, P-wave duration and spontaneous termination of AF episodes may be reflected by the absence of differences in conduction disorders found in our study. Our study is the first to use multiple clinical classifications to investigate differences in electropathology. However, our measurements were limited to SR. Measurements during programmed electrical stimulation and AF will elucidate direction and frequency dependent conduction disturbances and provide us with additional insights which are necessary for a classification based on severity of electropathology. Additionally, studies incorporating AF progression in relation to electropathology are needed. A classification based on severity of electropathology is requisite to improve on ablation therapies and to select the right patients for the various therapeutic strategies.

### 4.3. Conduction Disorders in Relation to AF Classification

Conduction disorders are generally assumed to play an important role, both in the commencement of AF and its maintenance [18]. Verheule et al. found lower atrial conduction velocities during slow pacing after cardioversion to SR in goats with pacemaker-maintained AF for 6 months compared to 3 weeks. After 6 months, goats had increased endomysial fibrosis, which is the probable explanation for this slowing of conduction during pacing [19]. Various endocardial electrophysiological studies in patients also reported slower conduction at BB and the LA in paroxysmal AF patients when compared to persistent AF patients [6,8,9,10]. However, BB was never measured directly, as mapping in these studies was performed at the endocardium. Lin et al. found a stepwise increase in SR LA activation time from patients without AF to paroxysmal AF, persistent AF and long-standing AF [9]. We also found a difference in LA activation time; however, this difference disappeared when correcting for possible confounding factors. Our study is the first high-resolution epicardial mapping study to show a difference in TAT and AT at BB during SR between paroxysmal and persistent AF after correction for patient characteristics. This prolongation of AT-BB reflects the advancement of interatrial conduction disorders in patients with persisting AF. As TAT remained significant, even after correction for left atrial enlargement which could also influence TAT, a prolonged AT of BB in patients with persistent AF also resulted in a prolonged TAT. Furthermore, persistent AF was an independent predictor for a longer TAT and AT at BB after correcting for AF prior to onset of the mapping procedure. This correction was made to adjust for the possible interference of electrical remodeling as measurements in some patients were performed shortly after electrocardioversion [20].

### 4.4. Bachmann’s Bundle in AF

In an experiment in 1971 by Waldo et al., a perpendicular lesion in BB was found to affect P-wave duration and produce an a-IAB-like P-wave [21]. Consequentially, it was assumed that BB plays a role in AF as a-IAB is associated with AF development and progression [22]. Additionally, Kumagai et al. found, using a sterile pericarditis canine model, that BB seemed to be critical in maintaining AF. During AF, BB was involved in many re-entry circuits and, after creating a full-thickness lesion in BB, AF was terminated and sustained AF could not be induced again [23]. At first glance these results may seem to contradict each other, as a full thickness lesion in BB inhibits AF induction while such a lesion also induces a-IAB which is associated with AF development and progression. However, in the case of a-IAB as seen on the ECG, conduction at BB is probably not blocked in the whole thickness of BB. In our study, more cCDCB lines and more perpendicularly orientated CB line parts at BB in patients with persistent AF accounted for a prolongation of SR activation time of BB. These conduction disturbances could lead to a-IAB-like changes on the ECG as conduction finds a faster alternative pathway leading to caudo-cranial activation of the LA. This was the case in our patients, as a P-wave duration of ≥120 ms or a-IAB on ECG correlated with AT-BB.

The pathophysiology behind the association between a-IAB and progression of AF is unclear. The link between persistence of AF and a longer AT at BB indicating more conduction disturbances can also not be concluded from our data. The higher amount of conduction disturbances indicated by a longer AT in patients with persistent AF found at BB could merely be the result of a higher susceptibility to AF remodeling of this location. However, the conduction disturbances could also contribute to a more stable AF, as suggested in prior studies [22,23]. AF stability could be the result of the extensive conduction disturbances at BB favoring re-entry in combination with the communicating role of BB linking the RA and LA. A more causal effect of conduction disturbances and the persistence of AF was indicated by the observed differences in conduction disturbances at BB in patients with a-IAB and/or a longer P-wave duration.

### 4.5. HATCH Score and Electropathology

The HATCH score was developed to predict progression of AF for patients with paroxysmal AF [12]. In follow-up studies, the success of the HATCH score was limited [24,25]. In our study, correlations were found between electrophysiological parameters and HATCH scores. Interestingly, one of these correlations was with AT at BB. However, the resulting correlation coefficients for HATCH scores with conduction parameters indicate only moderate correlations.

### 4.6. Limitations

There may be a bias in our study group as only AF patients in whom SR was restored after electrocardioversion were included. Furthermore, our study population is relatively small and it represents a specific subset of the AF population requiring cardiac surgery, which might not be representative of the general AF population. Our measurements were performed during SR, additional measurements during programmed electrical stimulation and AF will uncover potential direction and frequency-dependent conduction disturbances.

## 5. Conclusions

Using four different AF clinical classification methods, we did not find any conduction parameter during SR differentiating between AF subtypes in patients undergoing cardiac surgery. The observed differences in conduction parameters, mainly found at BB, were only moderately discriminatory. Future studies are needed to establish an objective classification for AF based on quantified features of electropathology in order to provide patient-tailored therapy of AF.

## Figures and Tables

**Table 1:** Baseline characteristics.

	Paroxysmal AF (*n* = 47)	Persistent AF (*n* = 24)	*p*
Age (years)	72.2 ± 7.3	71.0 ± 6.8	0.496
Gender: female	21 (44.7)	7 (29.2)	0.206
BMI (kg/m^2^)	27.3 ± 4.4	28.7 ± 4.3	0.198
Years since diagnosed AF	1.0 [0.3–5.0]	1.0 [0.4–5.5]	0.823
Diabetes mellitus	12 (25.5)	5 (20.8)	0.661
Hypertension	29 (61.7)	15 (62.5)	0.948
Dyslipidemia	13 (27.7)	6 (25.0)	0.811
History of myocardial infarction	10 (21.3)	3 (12.5)	0.521
AADs: Class I Class II Class III Class IV	1 (2.1) † 27 (57.4) 12 (25.5) 0	0 14 (58.3) 6 (25.0) 2 (8.3) †	0.845
Surgical indication: (i) VHD AVD AVD + CABG MVD MVD + CABG CABG	32 (68.1) 12 5 10 5 15 (31.9)	19 (79.2) 6 2 10 1 5 (20.8)	0.326 ‡
Left ventricular EF: Normal (≥50%) Mild impairment (40–49%) Moderate impairment (30–39%) Severe impairment (≤30%)	39 (83.0) 5 (10.6) 2 (4.3) 1 (2.1)	14 (58.3) 6 (25.0) 4 (16.7) 0	0.101
LA enlargement §	32/43 (74.4%) ¶	21/24 (87.5%) ¶	0.207
LAVI (mL/m^2^)	41 (33–56) (*n* = 31) ¶	44 (38–50) (*n* = 18) ¶	0.568

AAD: antiarrhythmic drugs; AVD: aortic valve disease; BMI: body mass index; CABG: coronary artery bypass graft; EF: ejection fraction; LAVI: left atrial volume index; MVD: mitral valve disease; (i)VHD: (ischemic) valvular heart disease; † these patients also used another class of AADs; ‡ comparison between (i)VHD and CABG; § left atrial enlargement was defined as LAVI > 28 mL/m^2^; ¶ indicates missing data.

## Data Availability

Data supporting the findings of this study is available on reasonable request from the corresponding author.

## Supplementary Materials

The following are available online at https://www.mdpi.com/article/10.3390/jcm10132846/s1: Table S1, prevalences of CD, CB and cCDCB per location; Table S2, multivariate analysis; Table S3, ROC curve AUC values.
