# Supplementary material for: Conduction Disorders during Sinus Rhythm in Relation to Atrial Fibrillation Persistence
Source: J Clin Med. 2021 Jun 27;10(13):2846. doi: 10.3390/jcm10132846 (PMC8269325; doi:10.3390/jcm10132846)
Supplement: Supplementary file 1 [file jcm-10-02846-s001.zip › jcm-1211483-supplementary.pdf]

Supplemental Materials

Table S1. Prevalences of CD, CB and cCDCB per location.

		PAF	persAF	*p*
**CD**	*RA*	3.6% [2.4–4.6]	3.1% [2.3–4.1]	0.206
	BB	5.8% [3.6–7.9]	6.0% [4.5–8.2]	0.668
	LA	2.7% [1.5–3.6]	3.1% [1.3–3.5]	0.743
	PV	4.1% [2.6–5.7]	2.9% [1.5–4.2]	0.161
**CB**	*RA*	3.1% [1.3–4.6]	2.7% [2.1–4.1]	0.923
	BB	4.9% [2.5–8.4]	5.0% [3.1–10.0]	0.668
	LA	1.2% [0.1–3.8]	1.8% [0.4–4.2]	0.230
	PV	1.6% [0.4–3.7]	1.1% [0.5–3.5]	0.803
**cCDCB**	*RA*	3.2% [1.6–5.7]	2.9% [1.9–5.1]	0.593
	BB	6.3% [2.7–11.8]	7.3% [5.7–13.0]	0.348
	LA	1.4% [0.1–4.0]	2.3% [0.6–4.6]	0.399
	PV	3.2% [0.5–5.8]	1.9% [1.1–4.6]	0.637

CD = conduction delay; CB = conduction block; cCDCB = continuous conduction delay and block; PAF = paroxysmal AF; persAF = persistent AF .

Table S2. Multivariate analysis.

	**TAT** **β [95% CI]**	***p***	(10log) **AT BB*** β [95% CI]	*p*	(10log) **AT LA*** β [95% CI]	*p*
AF type (persistent)	25.04 [2.37–47.71]	*0.031*	0.180 [0.048–0.312]	*0.008*	0.057 [−0.049–0.162]	0.286
Age	0.65 [−0.51–1.81]	0.269	0.008 [0.001–0.015]	*0.026*	0.005 [−0.001–0.011]	0.086
Gender (Male)	−0.74 [−17.98–16.49]	0.932	−0.108 [−0.203– −0.007	*0.036*	−0.027 [−0.107–0.052]	0.493
BMI	1.43 [−0.55–3.40]	0.153	0.007 [−0.005–0.018]	0.239	0.006 [−0.003–0.015]	0.214
Surgical indication (CABG)	−15.31 [−34.28–3.67]	0.112	−0.035 [-0.144–0.073]	0.515	0.013 [−0.076–0.102]	0.767
LA enlargement	11.78 [−9.05–32.61]	0.262	−0.006 [-0.131–0.118]	0.919	0.056 [-0.037–0.150]	0.234
AF prior to mapping	−18.76 [−42.59–5.08]	0.120	−0.097 [−0.233–0.038]	0.157	0.101 [−0.010–0.212]	0.074

Multivariate analysis with age, gender, BMI, diabetes mellitus, hypertension, dyslipidemia, surgical indication (val-vular with or without CABG or CABG), LAVI and AF prior to mapping as covariates to AF type. *For these parameters log transformation was performed, therefore for each unit change in X (e.g. the step from paroxysmal to persistent AF patients, Y (AT-BB or AT-LA) changes 10 ^ β; TAT = total activation time; BB = Bachmann's Bundle; BMI = body mass index; LAVI = left atrial volume-index; AT = activation time.

Table S3. ROC curve AUC values.

Clinical classification	Location	Conduction parameter	AUC [95% CI]
**PAF vs. persAF**	*BB*	*n* cCDCB lines	0.650 [0.513–0.786]
		n perpendicular CB line parts	0.650 [0.513–0.788]
		AT	0.681 [0.544–0.818]
	x	TAT	0.665 [0.523–0.807]
**ST vs. NST**	*BB*	AT	0.653 [0.521–0.786]
	x	TAT	0.689 [0.562–0.817]
**P-wave duration ≥ 120 ms**	*RA*	AT	0.722 [0.576–0.867]
	BB	%CB	0.717 [0.571–0.863]
		%cCDCB	0.670 [0.516–0.824]
		Length longest CB line	0.715 [0.565–0.866]
		Length longest cCDCB line	0.694 [0.537–0.851]
		AT	0.749 [0.608–0.890]
	PV	Length cCDCB lines	0.693 [0.529–0.857]

AT = activation time, AUC = area under the curve, BB = Bachmann's Bundle, CI = confidence interval, LA = left atrium, *n* = number, NST = No spontaneous termination of AF episodes, PAF = paroxysmal AF, persAF = persistent AF, RA = right atrium, ST = spontaneous termination of AF episodes.
